# *Leishmania Major* Centrin Gene-Deleted Parasites Generate Skin Resident Memory T-Cell Immune Response Analogous to Leishmanization

**DOI:** 10.3389/fimmu.2022.864031

**Published:** 2022-03-28

**Authors:** Nevien Ismail, Subir Karmakar, Parna Bhattacharya, Telly Sepahpour, Kazuyo Takeda, Shinjiro Hamano, Greg Matlashewski, Abhay R. Satoskar, Sreenivas Gannavaram, Ranadhir Dey, Hira L. Nakhasi

**Affiliations:** ^1^ Division of Emerging and Transfusion Transmitted Diseases, Center for Biologics Evaluation and Research (CBER), Food and Drug Administration (FDA), Silver Spring, MD, United States; ^2^ Laboratory of Clinical Hematology, Center for Biologics Evaluation and Research (CBER), Food and Drug Administration (FDA), Silver Spring, MD, United States; ^3^ Department of Parasitology, Institute of Tropical Medicine (NEKKEN), Nagasaki University, Nagasaki, Japan; ^4^ Department of Microbiology and Immunology, McGill University, Montreal, QC, Canada; ^5^ Department of Pathology and Microbiology, Ohio State University, Columbus, OH, United States

**Keywords:** Leishmania major, live attenuated Leishmania vaccine, tissue resident memory T cells, leishmanization, Granzyme B, cytokines

## Abstract

Leishmaniasis is a vector-borne parasitic disease transmitted through the bite of a sand fly with no available vaccine for humans. Recently, we have developed a live attenuated *Leishmania major centrin* gene-deleted parasite strain (*LmCen^-/-^*) that induced protection against homologous and heterologous challenges. We demonstrated that the protection is mediated by IFN (Interferon) γ-secreting CD4^+^ T-effector cells and multifunctional T cells, which is analogous to leishmanization. In addition, in a leishmanization model, skin tissue-resident memory T (TRM) cells were also shown to be crucial for host protection. In this study, we evaluated the generation and function of skin TRM cells following immunization with *LmCen^-/-^* parasites and compared those with leishmanization. We show that immunization with *LmCen^-/-^* generated skin CD4+ TRM cells and is supported by the induction of cytokines and chemokines essential for their production and survival similar to leishmanization. Following challenge with wild-type *L. major*, TRM cells specific to *L. major* were rapidly recruited and proliferated at the site of infection in the immunized mice. Furthermore, upon challenge, CD4^+^ TRM cells induce higher levels of IFNγ and Granzyme B in the immunized and leishmanized mice than in non-immunized mice. Taken together, our studies demonstrate that the genetically modified live attenuated *LmCen* ^-/-^ vaccine generates functional CD4^+^ skin TRM cells, similar to leishmanization, that may play a crucial role in host protection along with effector T cells as shown in our previous study.

## Introduction

Leishmaniasis is a vector-borne neglected tropical disease endemic in tropical and subtropical regions of the world. It is caused by infection with different species of the protozoan parasite *Leishmania* and transmitted by the bites of infected sand fly (1–4). Currently, there is no approved human vaccine against leishmaniasis, and existing treatment options are suboptimal because of the development of drug resistance and coinfections with HIV and other endemic diseases (3, 5, 6). Leishmanization, a process by which a small inoculum of *Leishmania major* parasites is injected into the skin to acquire protection against infection, was previously used in several countries in the Middle East and former Soviet Union (7). However, the practice has been discontinued because of safety concerns. Recently, using CRISPR (Clustered regularly interspaced short palindromic repeats) gene editing, we have generated a centrin gene-deleted live attenuated *L. major* strain, *LmCen^-/-^* (8). We demonstrated that *LmCen^-/-^* parasites do not cause lesions but have the ability to mount an immunological response that is protective against both cutaneous and visceral leishmaniasis in animal models that mimic human disease (8, 9). We have shown that both *LmCen^-/–^* immunized and healed mice from primary infection representing leishmanization generated comparable protective immunity against challenge with *LmWT* parasites. The protective immunity was due to multifunctional (IFNγ^+^ IL2^+^ TNFα^+^) CD4^+^ T cells as well as IFN-γ−secreting CD4^+^ T-effector cells (8).

Recent studies have suggested that memory T cells that accumulate in tissues, termed tissue-resident memory T (TRM) cells, play a crucial role in maintaining long-term protective immunity in skin, lungs, or any other mucosal organs against viral pathogens and allergens (10–13). The CD4^+^ and CD8^+^ TRM cells are identified by the expression of CD69 and CD103 in both mouse and human tissues (14, 15). It has been shown that skin CD69^+^ CD103^+^ TRM cells exhibit more effector function compared to CD69^+^ CD103^-^ (14). TRM cells can also be generated by vaccination, particularly live attenuated viral vaccines appear to be more effective than killed or subunit vaccines for inducing TRM cells (16–18).

Previous studies have shown that after the resolution of infection with wild-type *L. major* parasites, i.e., leishmanization, the skin of healed mice harbors CD4^+^ TRM cells, and the activity of these cells is important for optimal immunity against reinfection with *Leishmania* (19). The TRM cells persist in the absence of circulating *Leishmania*-specific T cells and rapidly recruit inflammatory monocytes and *Leishmania*-specific T effector cells to the site of infection and contribute to protective immunity (19, 20). In addition, intradermal delivery of a DNA vaccine for *Leishmania* was also found to generate long-lasting skin TRM cells that contributed to protective immunity against *L. major* challenge (21). Therefore, TRM cells are an excellent target for vaccine development (22). In this study, we show that intradermal immunization with genetically modified live attenuated *LmCen^-/-^* parasite vaccine generates CD4^+^ TRM cells in the skin of C57BL/6 mice comparable to leishmanization. Generation of CD4^+^ TRM cells in the skin by *LmCen^-/-^* was enabled by the expression of cytokines, chemokine receptors, and transcription factors as shown in previous studies (23). Following challenge with wild-type *L. major*, TRM cells specific to *L. major* were rapidly recruited and proliferated at the site of infection in the immunized mice and induced higher levels of IFN-γ and Granzyme B in the immunized and leishmanized mice than non-immunized mice. Therefore, the protection induced by *LmCen^-/-^* against infection with virulent *L. major* parasites might be, in part, due to rapid recruitment, proliferation, and induction of Th1 response and cytotoxic response (Granzyme B) by CD4^+^ TRM cells at the site of infection in addition to the IFNγ-secreting CD4+ T effector cells and multifunctional T cells (8). These observations suggest that intradermal immunization with *LmCen^-/-^* induces a protective response similar to leishmanization albeit a safer alternative to leishmanization.

## Materials and Methods

### Ethics Statement

The animal protocol for this study has been approved by the Institutional Animal Care and Use Committee at the Center for Biologics Evaluation and Research, US Food and Drug Administration (FDA) (ASP 1995#26). In addition, the animal protocol is in full accordance with “The guide for the care and use of animals as described in the US Public Health Service policy on Humane Care and Use of Laboratory Animals 2015.”

### *Leishmania* Strains and Culture Medium

*L. major* Friedlin (FV9) used in this study were routinely passaged into the footpads of BALB/c mice. Amastigotes isolated from infected lesions were grown in M199 medium, and promastigotes were cultured at 27°C in M199 medium (pH 7.4) supplemented with 10% heat-inactivated fetal bovine serum, 40 mM HEPES ((4-(2-hydroxyethyl)-1-piperazineethanesulfonic acid) (pH 7.4), 0.1 mM adenine, 5 mg L^−1^ hemin, 1 mg L^−1^ biotin, 1 mg L^−1^ biopterin, 50 U ml^−1^ penicillin, and 50 μg ml^−1^ streptomycin. Cultures were passaged in fresh medium at a 40-fold dilution once a week. The wild-type *L. major centrin* gene-deleted *LmCen* ^−/−^ (Friedlin strain) promastigotes were cultured as previously described (8).

### Mouse Infection and Immunization

Female 6- to 8-week-old C57BL/6 (Jackson labs) or IFNγ/Thy1.1 knock-in mice (24) were immunized or infected, in the upper right flank, with 2 × 10^6^ total stationary phase promastigotes of *LmCen^−/−^* or *LmWT* parasites, by intradermal needle injection, in 10 μl PBS. IFNγ/Thy1.1 knock-in mice were provided by C. Weaver (University of Alabama, Birmingham, AL, USA). After 15 weeks of infection/immunization, healed and immunized mice were challenged in the distal flank skin with 2 × 10^6^ total stationary phase *LmWT* promastigotes intradermally by needle inoculation.

### Flow Cytometric Analysis

T cells were isolated from the skin using the following protocol: after euthanasia, flanks were shaved and 1 cm^2^ of the flank skin was collected, then chopped and incubated in collagenase P (2 mg/ml; Roche Diagnostics) and DNAase in 10% FBS complete RPMI media at 37°C for 120 min. Tissue was then homogenized in MACS C tubes using gentleMACS Dissociator (Miltenyi Biotec) for 1 min. To obtain a single-cell suspension, tissue homogenate was strained through 100-µm and 70-µm nylon strains (Miltenyi Biotec). Cells were stained with antibodies, and their expression of phenotypic markers was determined by flow cytometry using BD LSR Fortessa (BD Biosciences) and analyzed with FlowJo (Treestar). We used the following antibodies: anti-mouse CD3 (17A2), CD44 (IM7), Thy-1.1 (HIS51), and Granzyme B (NGZB) from Thermo Fisher Scientific; anti-mouse CD4 (RM4-5), CD8a (53-6.7), CD62L (MEL-14), CD69 (H1.2F3), CD103 (2E7); anti-BrdU (B24) from BD Biosciences. Live cells were discriminated with a fixable LIVE/DEAD fixable blue dead cell stain (Thermo Fisher Scientific). Cell number, when indicated, was calculated as an absolute number per 10^6^ total acquired cells.

### Quantification of Gene Expression by qPCR

Cytokine expression from mouse skin tissues was determined by real-time PCR at the indicated time points. Briefly, total RNA was extracted from flank skin using PureLink RNA Mini kit (Ambion). Aliquots (300 ng) of total RNA were reverse transcribed into cDNA by using random hexamers from a high-capacity cDNA reverse transcription kit (Applied Biosystems). TaqMan gene expression Master Mix (Applied Biosystem) was used to determine the cytokine (**Table S1**) expression levels in a CFX96 Touch Real-Time System (Bio-Rad, Hercules, CA). The data were analyzed with CFX Manager Software. The expression levels of genes of interest were determined by the 2^-ΔΔCt^ method; samples were normalized to GAPDH expression and determined relative to expression values from naive animals.

### Histological and Immunohistochemical Staining

Mouse flanks were shaved before tissue harvesting. Flank skin was fixed in fixative solutions (10% buffered formalin phosphate solution). Paraffin-embedded sections were stained with H&E. Immune staining was also done using an Anti-Granzyme B Ab (EPR22645-206) from Abcam. All the histochemical and immunohistochemical staining was done by Histoserv (Gaithersburg, MD, USA). Stained sections were analyzed under a Keyence digital microscope (Keyence Corporation of America). For immunofluorescence, unfixed mouse skins were embedded in OCT compound embedding medium (Tissue-Tek) and cut into 10-µm sections for immunohistochemistry. Frozen sections were fixed with cold methanol for 5 min. Sections were blocked with 10% normal donkey serum and incubated with anti-CD3 (EPR20752), anti-CD69 (H1.2F3), and CD103 (AP-MAB0828) antibodies, from Abcam, overnight at 4°C. After washing with PBS, anti-rat, anti-rabbit, and anti-hamster secondary antibodies conjugated with Alexa Fluor 488, 594, and 647 (Jackson ImmunoResearch), respectively, were applied for 1 h at room temperature, washed and followed with Hoechst 33258 nuclear counterstaining, and mounted with Fluoromount-G. These slides were examined with a Leica SP8 confocal microscope.

### *In Vivo* Bromodeoxyuridine Treatment

Mice were injected intraperitoneally with 2 mg of BrdU per day, for 3 days, with treatment starting on the day of challenge with *LmWT* parasites. BrdU incorporation was measured with a BrdU flow kit (BD Biosciences), and the proportion of BrdU^+^ cells was measured by flow cytometry and analyzed by FlowJo software as indicated in **Supplementary Figure S4**.

### Adoptive Transfer of T Cells

For cell recruitment studies, T cells from spleens of 4 weeks *LmCen^-/-^* immunized mice were isolated using mouse Pan T cell isolation kit (Miltenyi Biotec) according to the manufacturer’s protocol. The cells were then stained with Invitrogen CellTrace Far Red Cell Proliferation Kit (Fisher Scientific). The stained cells (30 × 10^6^/mouse) were transferred intravenously (i.v.) into naive recipient mice. After 24 h, recipient mice were then challenged in the flank skin with *L. major WT* parasite (2 × 10^6^/mouse). Skin from challenged flanks was collected at 48 h post-challenge and prepared for flow cytometry analysis.

### Statistical Analysis

Statistical analysis of differences between means of groups was determined using a two-tail unpaired t test. All proportional numerical values provided in the text and figure legends were written as the mean ± SEM. All statistical analyses were done in Prism 7.0 (GraphPad). All experiments were performed at least two times, with similar results obtained each time.

## Results

### Immunization With *LmCen* ^-/-^ Generates CD4^+^ TRM Cells in the Skin

**Figure 1:**
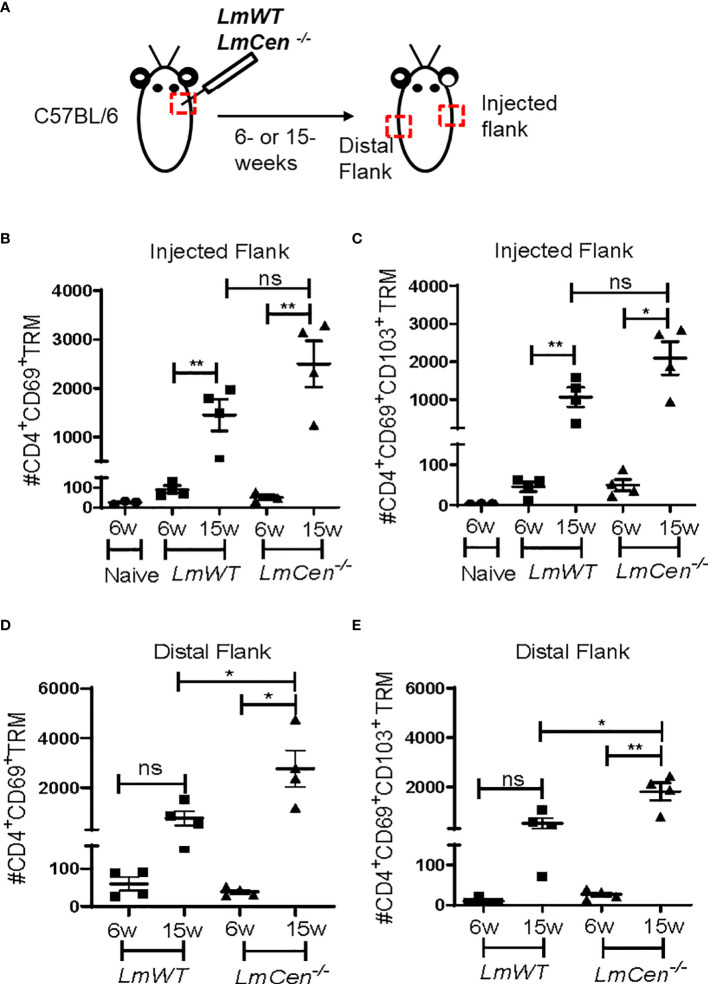
Immunization with *LmCen* ^-/-^ generates CD4^+^ TRM cells in the skin. Mice were injected, intradermally, with either *LmCen^-/-^* or *L. major* wild-type (*LmWT)* parasites in the right flank, and TRM cell population was assessed at 6 and 15 weeks post-injection by flow cytometry from both right (injected flank) and left flank (distal flank) marked by the red box. Baseline TRM population was measured in flank skin of non-immunized mice. **(A)** Schematic plan of injection site and experimental time points. **(B, C)** TRM cell population in the injected flank. **(B)** CD4^+^CD69^+^ TRM cells and **(C)** CD4^+^CD69^+^CD103^+^ **. (D, E)** TRM cell population in the distal flank. **(D)** CD4^+^CD69^+^ TRM cells and **(E)** CD4^+^CD69^+^CD103^+^ collected from *LmWT* and *LmCen^-/–^* injected mice at 6 and 15 weeks post-injection. The Y axis represents the number of TRM cells per 10e6 total cells acquired. Results are representative of one of two independent experiments with n = 3–4 mice per group. Bars represent the means with SEM in each group. Statistical analysis was performed by unpaired two-tailed t-test (**p < 0.009, *p < 0.05, ns, not significant).

Previous studies of the leishmanization model have shown that resolution of acute infection with *LmWT* parasites (12–20 weeks post-infection) is accompanied by the formation of CD4^+^ TRM cells that contribute to protective immunity against virulent challenge (19, 20). To investigate if immunization with *LmCen^-/-^* parasites would similarly lead to the formation of TRM cells, we injected C57BL/6 mice intradermally in the flank skin with 2 × 10^6^ stationary phase of either *LmCen^-/-^* or *LmWT* parasites and monitored TRM populations in both groups (**Figure 1A**). Next, we evaluated both CD4^+^CD69^+^ as well as CD4^+^CD69^+^CD103^+^ TRM cell populations in the injected and distal flanks at both 6 and 15 weeks post-infection/immunization (**Figures 1B–E**). We identified TRM population as CD3^+^CD4^+^CD44^+^CD62L^-^CD69^+^ and CD3^+^CD4^+^CD44^+^CD62L^-^CD69^+^CD103^+^ (**Supplementary Figure S1**). We observed that most of the TRM cells expressing CD69 are also expressing CD103 (**Supplementary Figure S1**). The purpose of evaluating the generation of TRMs at 6 weeks was to establish a baseline for the response observed at the 15-week time point. There were very few CD4^+^CD69^+^ as well as CD4^+^CD69^+^CD103^+^ TRM cells in the injected flank at 6 weeks PI in both *LmCen^-/–^* and *LmWT*-infected mice (**Figures 1B, C** **)**. However, in both *LmCen^-/–^* immunized mice and *LmWT*-infected mice (healed mice), we observed significantly higher numbers of both CD4^+^CD69^+^ and CD4^+^CD69^+^CD103^+^ TRM in the injected flank at 15 weeks compared to 6 weeks PI or compared to non-immunized mice (**Figures 1B, C** **)**. Our data indicate that, in the injected flank, at 15 weeks of PI, there was no significant difference between *LmCen^-/–^* immunized mice and *LmWT*-infected mice (**Figures 1B, C** **)**. However, at the distal flank, there was a significant increase of both CD4+CD69+ and CD4+CD69+CD103+ TRM cells in *LmCen^-/–^* immunized group compared to *LmWT* healed group at 15 weeks of PI (**Figures 1D, E** **)**. We also noted that the frequencies of CD4^+^ TRM cells in the injected and distal flanks are comparable (**Figures 1B–E**), indicating that the TRM cells were present in more or less uniform density throughout the skin of immunized animals. Taken together, these data indicate that immunization with *LmCen^-/-^* parasites generates CD4^+^ TRM cells in the injected as well as distal sites of the skin of mice.

### Expression Profile of Cytokines and Chemokine Receptors Supporting Tissue-Resident Memory T Cell Generation at the Injected and Distal Flank Skin

**Figure 2:**
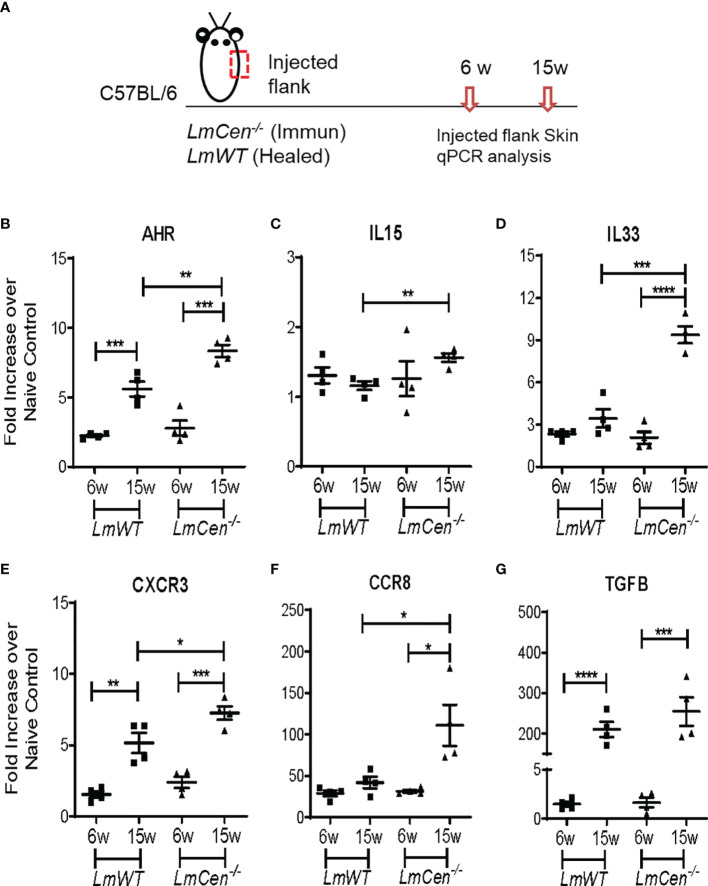
Expression profile of cytokines and chemokine receptors supporting TRM cell generation at the injected flank skin. Mice were injected with *LmCen^-/-^* or *LmWT*, intradermally, in the right flank. The expression profile of indicated genes from the injected flank skin was assessed at 6 and 15 weeks (6w and 15w) post-injection by qPCR. **(A)** Schematic plan of the experimental time points and injection site. **(B–G)** Expression of different transcripts, **(B)** AHR**, (C)** IL15, **(D)** IL33**, (E)** CXCR3, **(F)** CCR8, and **(G)** TGFB, at indicated time points. To determine the fold expression of each gene, 2^-ΔΔCT^ method was employed. The data were normalized to GAPDH expression and shown as the fold change relative to age-matched naive mice. Results are representative of one of two independent experiments, with total 4 mice per group. Bars represent the means with SEM in each group. Statistical analysis was performed by unpaired two-tailed t test (*p < 0.05, **p < 0.009, ***p < 0.0005).

To investigate the immunological milieu that supports TRM cells, we determined the expression of several cytokines and chemokine receptors (AHR, IL15, IL33, CXCR3, CCR8, and TGFβ) known to support the formation, survival, and homeostasis of TRM cells (24). RNA was isolated from the injected flank skin of the *LmCen^-/–^* or *LmWT*-injected mice, and the indicated analytes were assayed by q-PCR (**Figure 2A**). In the injected flank of *LmCen^-/–^* immunized mice, the expression of AHR, IL33, CXCR3, CCR8, and TGFB was significantly upregulated at 15 weeks PI compared to 6 weeks PI (**Figures 2B, D–G**). Our data indicate that the expression of AHR, IL15, IL33, CXCR3, and CCR8 was significantly higher in the *LmCen^-/–^* immunized mice compared to *LmWT* healed mice at 15 weeks PI (**Figures 2B–F**). In the *LmWT* healed group, only AHR, CXCR3, and TGFB were significantly upregulated at 15 weeks (**Figures 2B, E, G** **)**. Since we observed increased population of CD4+ TRM cells in the distal flank (**Figures 1D, E** **)**, we wanted to investigate if such increase in TRM cell population was supported by differential expression of cytokines or chemokine receptors in the distal flank as well. In the distal flank, of both *LmCen^-/–^* immunized mice and *LmWT* healed mice, the expression of AHR, IL33, and CXCR3 was significantly upregulated at 15 weeks PI compared to 6 weeks PI (**Supplementary Figures S2A, B, D, E**). However, there was no difference in the expression of IL15 at 15 weeks PI compared to 6 weeks PI in both immunized and *LmWT* healed mice (**Supplementary Figure S2C**).

Overall, these results suggest that the cytokine milieu in the skin of *LmCen^-/–^* immunized mice is conducive to support the formation, survival, and homeostasis of TRM cells at both injected and distal sites. Furthermore, our results suggest that immunization with *LmCen^-/-^* parasites results in slightly higher cytokine milieu in the skin compared to leishmanization.

### *LmWT* Challenge Leads to Rapid Accumulation of Tissue-Resident Memory T Cells in the Skin of Immunized or Healed Mice

**Figure 3:**
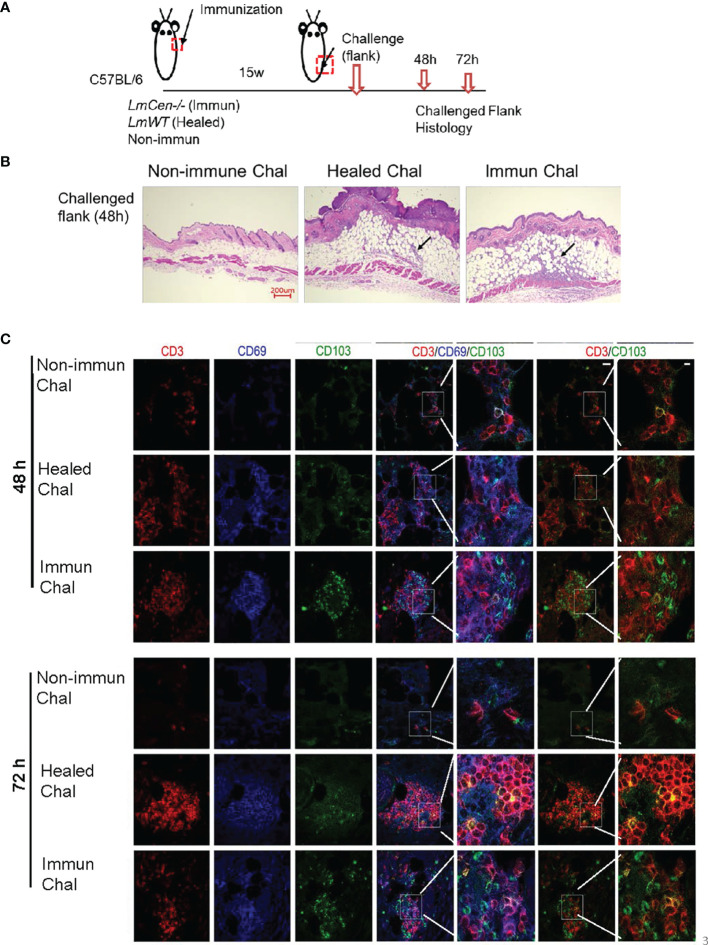
*LmWT* challenge leads to rapid accumulation of TRM cells in the skin of immunized or healed mice. Fifteen-week (15w) immunized and healed mice and non-immunized control mice were challenged in the flank skin with *LmWT* virulent parasite. Skin from the site of challenge was collected at 48 and 72 hours (48h and 72h) post-challenge. Tissue infiltration and TRM cells were analyzed by immunofluorescence and H&E staining. **(A)** Schematic plan of the experimental time points. **(B)** H&E staining of skin tissue at the site of challenge 48 h post-challenge. Black arrow indicates cellular infiltration at the challenge site. **(C)** Expression of CD3 (red), CD69 (blue), and CD103 (green) in the flank skin of non-immunized control, healed, and immunized mice, at the site of challenge, following 48 and 72 h post-challenge. Scale bar is 25 µm. Results are representative of one independent experiment, repeated 3 times, with 3 mice per group.

To study *Leishmania*-specific TRM cell recall response in the skin, non-immunized, *LmCen^-/–^* immunized, or healed mice were challenged with *LmWT* parasites 15 weeks PI at a site different from the initial immunization or infection site (**Figure 3A**). At 48 and 72 h post-challenge, skin from the challenge site was collected for histology and immunohistochemistry (**Figure 3A**). The challenged flank skin stained with H&E clearly revealed a robust accumulation of cells in both *LmCen^-/–^* immunized mice and healed mice compared to non-immunized mice at 48 h post-challenge (**Figure 3B**). Furthermore, TRM cells before (**Supplementary Figure S3A**) and after 48 and 72 h post-challenge were identified by staining for CD3, CD69, and CD103 **(** **Figure 3C** **).** Before challenge, we observed a few CD69+CD103+ cells in the skin of immunized or healed mice (**Supplementary Figure S3B**). In the immunized mice after challenge with *LmWT* parasites, immunohistochemistry-stained section of the challenged site showed a rapid increase of CD103+ cells within 48 h compared to non-immunized or healed mice that persisted until 72 h (**Figure 3C**). This increase was also observed in CD3^+^CD103^+^ cells, as revealed in the overlay of red (CD3^+^) and green (CD103^+^) fluorescence images (**Figure 3C** **, inset**). In healed mice, the accumulation of TRM cells (CD3^+^ CD103^+^) after challenge with *LmWT* parasites was distinct from non-immunized mice only at 72 h post-challenge (**Figure 3C**). Non-immunized challenged mice showed little accumulation of TRM cells at the site of challenge that indicates that the rapid accumulation of TRM cells at the site of challenge is a recall response to *LmWT* challenge infection.

### Tissue-Resident Memory T Cells From *LmCen^-/–^* Immunized Mice Proliferate Locally Following Challenge With Virulent *LmWT* Parasites

**Figure 4:**
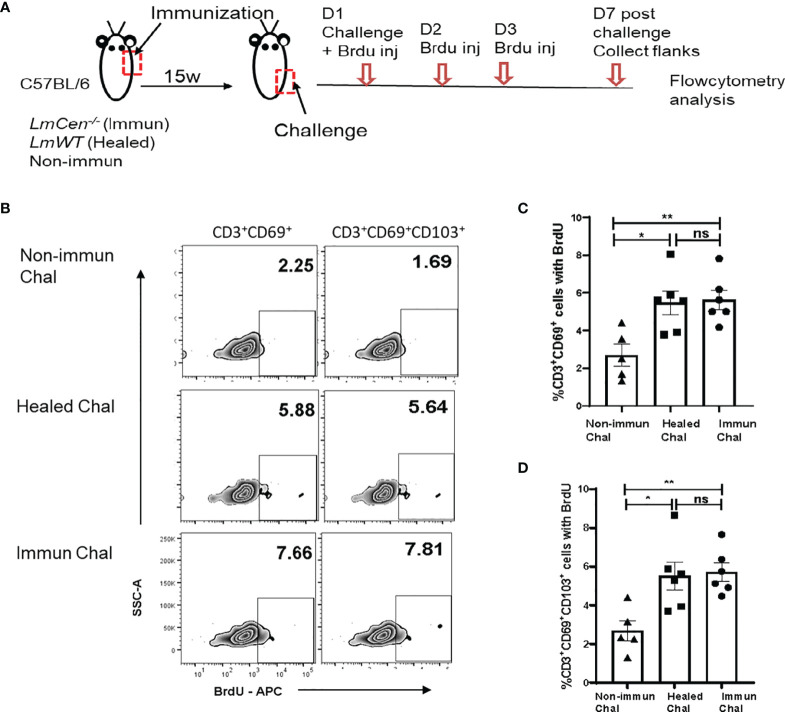
TRM cells from *LmCen^-/–^* immunized mice proliferate locally following challenge with virulent *LmWT* parasites. Fifteen**-**week (15w) immunized and healed mice were challenged with *LmWT* parasites and injected with BrdU, as described in the *Materials and Methods*. Mice were euthanized, and flank skins were collected 7 days post-challenge and analyzed for BrdU-positive cells. **(A)** Schematic plan of the experiment. **(B)** Representative BrdU staining on skin CD69^+^ (single positive) and CD69^+^CD103^+^ (double positive) TRM cells 7 days post-*LmWT* challenge. The numbers represent the percentage of gated population as a ratio of total parent population (CD69^+^ or CD69^+^CD103^+^, **C, D**, respectively). Panels **(C, D)** show the proportion of skin CD69^+^ and CD69^+^CD103^+^ TRM cells, respectively, with incorporated BrdU in the Non-immunized, Healed, and Immunized mice 7 days post-*LmWT* challenge. *p < 0.02, **p = 0.005, ns = not significant. Data shown are combined results from two independent experiments, n = 5–6. Results are mean ± SEM; statistical analysis was performed by two-tailed unpaired t-test. D, Days; Chal, Challenged; inj, Injection.

It was shown that TRM cells proliferate *in situ* after an antiviral recall response (25). To assess the proliferative capacity of TRM cells in response to *LmWT* challenge, immunized, healed, or non-immunized mice were treated with bromodeoxyuridine (BrdU) for three consecutive days starting on the day of challenge (**Figure 4A**). Seven days post-challenge, cells were isolated from the challenged flank skin to detect the proliferating TRM cells (identified by incorporated BrdU, i.e., BrdU^+^ cells). We excluded central memory T cells by gating on CD3^+^CD62L^low^ cells (**Supplementary Figure S4**). We observed that both immunized and healed mice had significantly higher proportion of CD3^+^CD69^+^BrdU^+^ (**Figures 4B, C** **)** and CD3^+^CD69^+^CD103^+^BrdU^+^ (**Figures 4B, D** **)** T cells compared to the non-immunized challenged group. The proportion of both CD3^+^CD69^+^BrdU^+^ and CD3^+^CD69^+^CD103^+^BrdU^+^ T cells from immunized challenged mice was similar to that observed in the healed challenged group (**Figures 4C, D** **)**. These results indicate that *LmCen^-/-^* immunization generates skin TRM cells that proliferate, *in situ*, in response to challenge with virulent *L. major* parasite.

It has been shown in the leishmanization model that TRM cells enhance T-cell recruitment to the site of challenge (19). In the current study, we wanted to investigate if TRM cells generated by *LmCen^-/-^* immunization have any role in the recruitment of *Leishmania*-specific T cells from circulation to the site of challenge. The T cells were collected from the spleens of *LmCen^-/–^* immunized mice and stained by CellTrace and injected intravenously (i.v.) into non-immunized, healed, or *LmCen^-/–^* immunized mice. The recipient mice were then challenged in the flank skin with *LmWT* parasites (**Supplementary Figure S5A**). To assess T-cell recruitment to the site of challenge, we assessed the percentage of CellTrace-positive T cells isolated from the challenged flank 48 h post-challenge (**Supplementary Figure S5B** **).** We observed that upon challenge, the skin of *LmCen^-/–^* immunized mice and healed mice had significantly higher proportion of CellTrace-positive T cells compared to non-immunized challenged mice that lack *L. major*-specific skin TRM cells (**Supplementary Figure S5C**). There was no difference in CellTrace-positive T-cell recruitment between *LmCen^-/–^* immunized and healed mice (**Supplementary Figure S5C**).

### Skin Tissue-Resident Memory T Cells From *LmCen^-/–^* Immunized Mice Produce IFNγ in Response to Challenge

**Figure 5:**
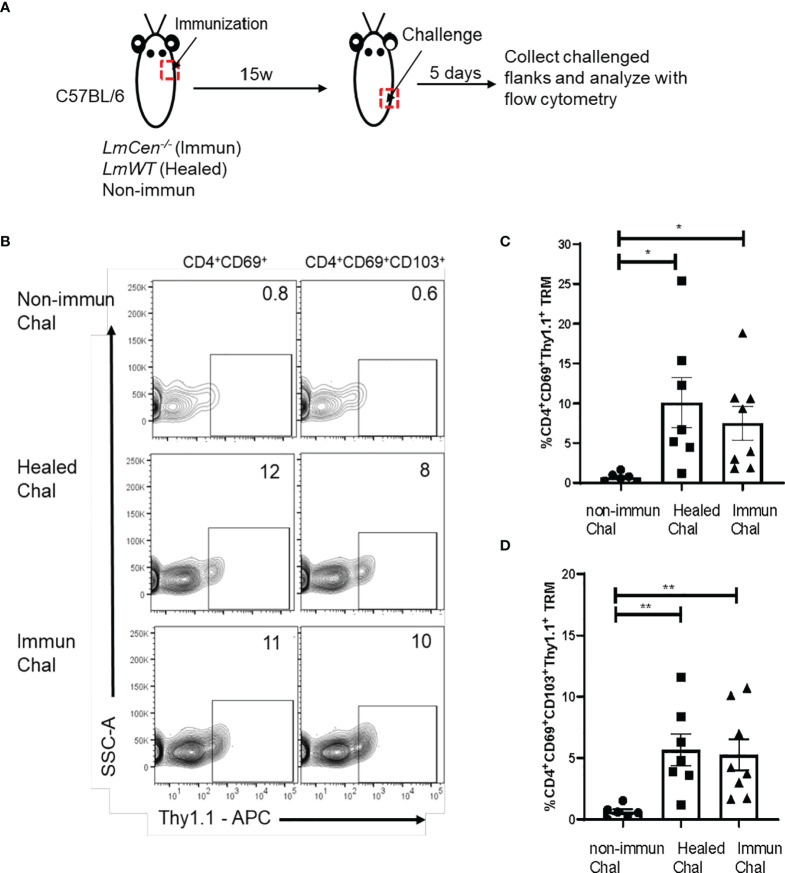
Skin TRM cells from *LmCen^-/–^* immunized mice produce IFNγ in response to challenge. Non-immunized, healed, and immunized IFNγ/Thy1.1 mice were challenged with *LmWT* parasite in the flank skin. T cells from challenged skin were isolated 5 days post-challenge, and cytokine production was assessed with flow cytometry analysis. **(A)** Schematic representation of the experiment. **(B)** Graph is representative of Thy1.1 staining on skin CD4^+^ TRM cells (CD3^+^CD4^+^CD44^+^CD62L^-^CD69^+^ and CD3^+^CD4^+^CD44^+^CD62L^-^CD69^+^CD103^+^). **(C, D)** Expression of Thy1.1 surface marker, representing IFNγ expression, was measured on **(C)** CD4^+^CD69^+^ and **(D)** CD4^+^CD69^+^CD103^+^ TRM cells. Y axis represents a portion of skin CD4^+^ TRM cells expressing IFNγ as a percentage of CD4^+^CD69^+^ TRM population. Data shown are combined results from two independent experiments with n = 6–8 mice per group. Bars represent the means with SEM in each group. Statistical analysis was performed by unpaired two-tailed t-test (**p < 0.007, *p < 0.02). Chal, Challenged; w, week.

We wanted to evaluate if TRM cells are capable of producing IFNγ upon challenge. To increase the sensitivity of detection of IFNγ, we used *IFNγ/Thy1.1* knock-in mice, where IFNγ-expressing cells could be identified by the surface expression of Thy1.1 (24). Non-immunized, immunized, and healed *IFNγ/Thy1.1* knock-in mice were challenged with virulent *LmWT* parasites in the lower flank skin (**Figure 5A**). Five days post-challenge, cells were isolated from the challenged site skin, and the expression of IFNγ on TRM cells was measured by flow cytometry without any *ex vivo* antigen restimulation and compared among the groups (**Figures 5A, B** **;** **Supplementary Figure S6**). We observed that both CD4^+^CD69^+^ and CD4^+^CD69^+^CD103^+^ TRM cells from immunized and healed mice showed significantly higher expression of IFNγ (represented by Thy1.1 expression) compared to that of non-immunized mice following challenge (**Figures 5C, D** **)**. Importantly, IFNγ expression by TRM cells was comparable between healed and immunized mice (**Figures 5C, D** **)**. Taken together these, data indicate that *Leishmania*-specific skin TRM cells, generated after immunization with *LmCen^-/-^* parasites, can mount a Th1 response upon challenge with virulent parasite.

### Tissue-Resident Memory T Cells From *LmCen^-/–^* Immunized Mice Exhibit Cytotoxic Function by Expressing Granzyme B

**Figure 6:**
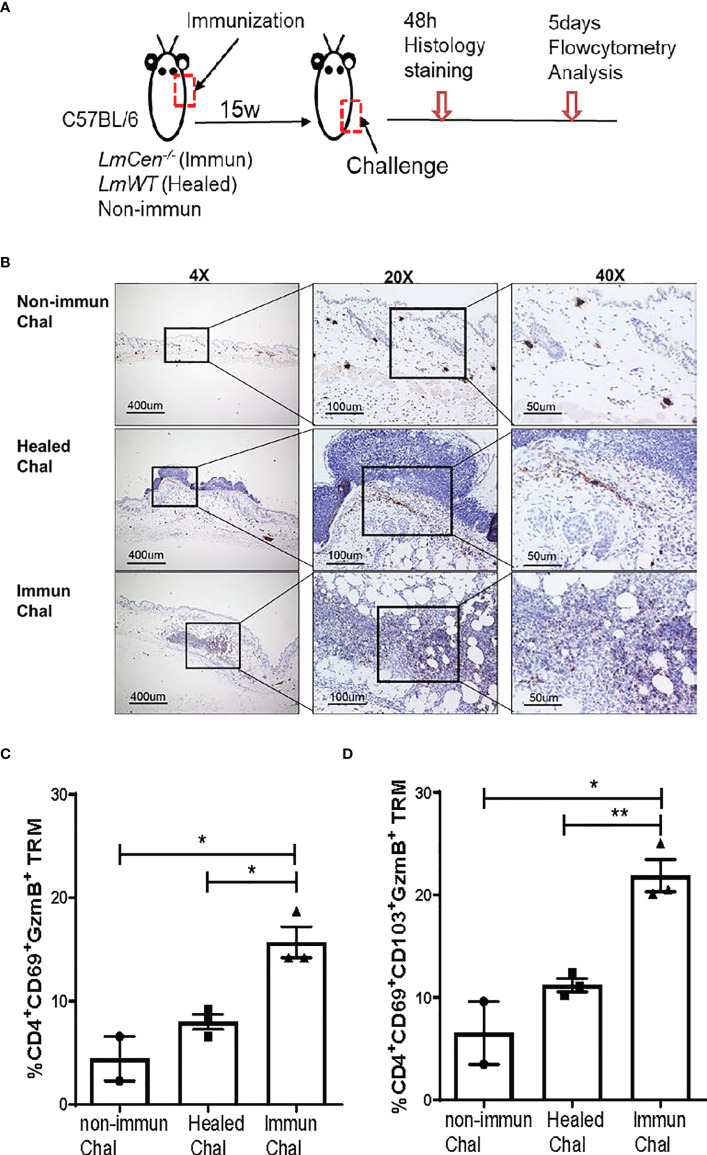
TRM cells from *LmCen^-/–^* immunized mice exhibit cytotoxic function by expressing granzyme B (GzmB). Fifteen-week 15w immunized and healed mice and non-immunized control mice were challenged in the flank skin with *LmWT* virulent parasites. **(A)** Schematic diagram of the site of injections. **(B)** Immunohistochemistry of the flank skin, at the challenge site, from 48 h challenged mice, stained with anti-granzyme B antibodies. **(C, D)** T cells from challenged skin were isolated 5 days post-challenge, and granzyme B production was assessed with flow cytometry analysis. **(C)** Portion of CD4 TRM cells, single positive (CD4^+^CD69^+^), expressing granzyme B **(D)** Portion of CD4 TRM cells, double positive (CD4^+^CD69^+^CD103^+^), expressing granzyme B Results are representative of one of two independent experiments with total 2–4 mice per group. Bars represent the means with SEM in each group. Statistical analysis was performed by unpaired two-tailed t-test (**p < 0.004, *p < 0.03). h, hours.

To investigate if the *LmCen^-/–^* induced TRM cells exhibit cytotoxic function following virulent infection, we challenged non-immunized, immunized, and healed mice in the flank skin with *LmWT* parasites (**Figure 6A**). We collected the flank skin 48 h post-challenge and stained the tissue with anti-granzyme B antibodies (**Figure 6A**). Immunohistochemistry staining showed that granzyme B expression is higher in the tissue of *LmCen^-/–^* immunized mice compared to healed and non-immunized mice following challenge (**Figure 6B**). The expression of granzyme B in non-immunized mice was minimal (**Figure 6B**). Next, we wanted to investigate if the cells producing granzyme B are indeed TRM cells. Non-immunized, immunized, and healed mice were challenged with *LmWT* parasites, and granzyme B production by TRM cells was assessed by flow cytometry. We found that a significant proportion of CD4^+^CD69^+^ TRM cells from the skin of *LmCen^-/–^* immunized mice produced granzyme B post-challenge, which was significantly higher compared to healed or non-immunized mice (**Figure 6C**). The proportion of granzyme B-producing CD4^+^CD69^+^CD103^+^ TRM cells was also significantly higher in the *LmCen^-/–^* immunized mice compared to healed and non-immunized mice following challenge (**Figure 6D**). Taken together, these results indicate that *LmCen^-/-^* immunization induces CD4^+^ TRM cells with a cytotoxic response after challenge, and such response is equivalent to healed infection.

## Discussion

Skin is the first line of defense against infection with vector-borne pathogens like *Leishmania* parasites. In addition to its physical, chemical, and microbiological barriers against pathogens, human skin harbors innate immune cells as well as a combination of resident and recirculating memory T cells with potent effector functions (14, 26). Upon infection, these cells induce a robust immune response against invading pathogens. In recent years, a T-cell lineage termed TRM cells has been identified as the first line of defense against viral infections entering the body at barrier sites, like the skin (27). Similarly, these cells have also shown to play an important role in host immunity against parasitic infections such as leishmaniasis and malaria (19, 20, 22). Specifically, it has been demonstrated that protection upon challenge with *L. major* parasites in healed (i.e., leishmanization) is mediated by the TRM cells that help recruit heterogenous cell populations including CD4^+^ T-effector cells and inflammatory monocytes to the site of infection and mediate parasite control (19, 20). Prior studies in leishmanization models also showed the critical role of IFNγ-secreting CD4^+^CD44^+^Ly6C^+^ T effector cells in protection against challenge by virtue of their capacity to mount microbicidal activities immediately after challenge (28, 29). However, since the presence of “ready-to-act” CD4^+^CD44^+^Ly6C^+^ T-effector cells requires a persistent infection of *Leishmania* parasites, achieving durable protection through safer vaccination methods makes TRM populations more desirable to target. Such vaccination may be achieved through live attenuated *Leishmania* strains that can persist at low levels in the immunized host yet lack virulence (8). Moreover, such vaccines may circumvent the unresolved issues associated with persistent infection of *Leishmania* observed in leishmanization models such as 1) indefinite maintenance of T-effector populations in the presence of concomitant immunity and 2) potential for T-cell exhaustion due to continuous exposure to persisting antigens as discussed previously (28). However, little is known about the role of TRM cells in vaccine immunity in general and particularly in genetically modified live attenuated vaccine candidates including *Leishmania.*

Recently, we have reported on using CRISPR-Cas9 technology to develop *centrin* gene-deleted *L. major* parasites (*LmCen^-/-^*). *LmCen^-/-^* parasites showed limited survival in the host and induced protective immunity comparable to leishmanization against both needle and sand fly challenge with a wild-type *L. major* as well as *L. donovani* infection (8, 9). We also demonstrated that upon challenge with *LmWT* parasites, both *LmCen^-/–^* immunized and healed mice generated a comparable CD4^+^Ly6C^+^IFNγ^+^ effector T-cell response (8), which has been shown to play a role in parasite killing upon reinfection (30). Since, in addition to CD4^+^ effector T cells, it was reported that skin resident CD4^+^ TRM cells are generated in response to *L. major* infection, after lesions are healed, and that they play an important role in protection against reinfection along with circulating memory T cells (19). In this study, we evaluated the generation and function of skin TRM cells following immunization with *LmCen^-/-^* parasites in mice and compared the response with mice that were healed after a primary *LmWT* infection. It is important to distinguish various T memory cell populations in characterizing the vaccine immunity. Since CD62L is a marker specific for central memory T cells, we have excluded this population while analyzing TRM cells. In this study, TRM cells were identified by the expression of both CD69 and CD103 (15), as it was previously shown that expression of both markers is necessary for the optimal formation and survival of TRM cells in the skin (31, 32). The skin of immunized and healed mice sampled at 15 weeks of post-infection time point showed a significantly higher population of TRM cells compared to naive control group, similar to previous studies (19, 20). It has been shown that TRM cells are not restricted to the original site of infection but have the capacity to disseminate throughout the skin (33, 34). Interestingly, in the *LmCen^-/–^* immunized group, we found that CD4^+^ TRM cells were significantly higher in numbers in the distal sites compared to healed mice, suggesting that *LmCen* ^-/-^ immunization is efficacious at inducing TRM cell populations. Previously, it has been shown that a population of skin resident CD4+ regulatory T cells (CD4+CD25+Foxp3+) also express CD103 (35, 36) and that CD103+ T regulatory (T Reg) cells play a crucial role in *Leishmania* infection-induced pathology (36). Since CD69+CD103+ T cells were observed at 15 weeks post-*LmCen^-/-^* immunization at which point we could not recover any viable parasites, it is unlikely that these cells are CD4+ T Reg cells that require the presence of persistent infection (36). Additionally, the residency of T Reg cells in the non-lymphoid organs in the absence of persistent infection is not fully understood (37, 38). Future studies will delineate the roles of T Reg and TRM cells in *LmCen^-/-^* vaccine-induced immunity.

The role of chemokine receptors in the formation and survival of skin TRM cells has been previously documented (39, 40). It has been shown that aryl hydrocarbon receptor (AHR) is required for long-term persistence of skin TRM cells (39, 41). We observed that *LmCen^-/-^* immunization induced significantly higher expression of AHR compared to healed mice. Similarly, IL15, IL33, and TGFβ are shown to be required for the development and maintenance of TRM cells in the skin following viral (Herpes Simplex Virus) infection (31, 42, 43). In our study, we observed significantly higher expression of these cytokines in the *LmCen^-/–^* immunized group as well as healed animals compared to non-immunized animals, suggesting that immunization with *LmCen* ^-/-^ induces an immune milieu that enables the generation of TRM cells, similar to leishmanization. Future studies will need to address the mechanisms by which these cytokines will help in the maintenance of long-term TRM cells in the skin of *LmCen^-/–^* immunized mice.

In localized infections, TRM cells are highly protective and modulate host immune response by 1) killing the pathogen-infected cells through direct lysis, 2) release cytokines that further enhance local recruitment of other innate and adaptive immune cells, and 3) proliferate *in situ* to maintain a stable population of protective TRM cells (25, 44). We investigated each of these functions in both immunized and healed mice in response to virulent *LmWT* challenge. Similar to studies in humans, where activated memory CD4^+^ T cells, termed CD4^+^ CTL cells, secreted a similar amount of granzyme B compared to memory CD8^+^ T cells (45), CD4^+^ TRM cells from the skin of *LmCen^-/–^* immunized mice produced granzyme B very early on upon challenge. These cells are mainly localized in peripheral tissue like the skin and were found to play an important role in antiviral immunity as studied by others (45, 46). Parasite-specific granzyme B production by human peripheral blood mononuclear cells (PBMCs) was found to be a good correlate of protection against *Leishmania* infection and could be a biomarker of vaccine-induced protection against human leishmaniasis (2, 47). Our study is the first to show granzyme B production by activated CD4^+^ TRM cells in response to *Leishmania* challenge in a mouse model illustrating the direct antimicrobicidal activities of CD4^+^ TRM cells in protection.

Toward characterizing the effector function from TRM populations, we analyzed cytokine responses in the skin following challenge. We observed pro-inflammatory cytokine, IFNγ, to be specifically expressed by TRM cells upon challenge with *LmWT* parasites. In addition, as early as 48 h post-challenge, we observed an abundant number of immune cells at the site of infection compared to non-immunized challenged mice. These cells included CD69^+^CD103^+^ TRM cells that progressively increased in number from 48 to 72 h post-challenge. As shown in previous studies, this increase could be due to the proliferation or recruitment from adjacent sites or from circulation to the site of infection (25). Accordingly, we have found that *LmCen^-/–^* specific TRM cells proliferate *in situ* and might play a role in recruiting *Leishmania* antigen-specific T cells from circulation in response to *LmWT* challenge. Consistent with the cytokine milieu that enables the production and maintenance of TRM population observed in *LmCen^-/-^* immunization, an equivalent TRM response between immunization and healed infection suggests that immunization with *LmCen^-/-^* is potent at inducing this population without causing pathology. A formal demonstration of the role of TRMs induced by *LmCen^-/-^* immunization in the recruitment of heterogeneous cell populations or local proliferation at the site of challenge will require further studies.

TRM cells have been shown to mediate effector functions similar to circulating T memory populations that have been studied extensively in various infection models. As TRM cells are located in the peripheral tissues, early activation and gaining of antimicrobial function are some of the crucial characteristics of these cells. Due to their effector activity, the TRM cells have been shown to control viral infections in various preclinical studies in mice in addition to the circulating memory cells (48, 49). Similarly, in non-human primates, the presence of TRM cells against Simian Immunodeficiency virus (SIVs) or Ebola virus was essential to control the viral load (50, 51). In *Leishmania*, where the rapidity of response is essential for protection against sand fly bite transmission, the presence of skin TRM cells along with effector cells can be the first line of defense keeping the parasites in check until the activation of central memory T cells takes place (30, 52). In the current study, we observed that within 48 h post-challenge, CD69^+^CD103^+^ TRM cells accumulated in the challenged site in *LmCen^-/–^* immunized mice, and these cells remained abundant at 72 h in the infected site. Consistent with previous studies, the TRM cells secreted IFNγ and granzyme B, indicating that their cytotoxic activities may be important in the protection observed in *LmCen* ^-/–^induced immunity.

Overall, our results establish that *LmCen^-/-^* immunization generates CD4^+^ TRM cells in the skin of immunized animals. Moreover, *LmCen^-/-^* immunization-induced immunity is comparable to that of healed mice as previously measured by effector T cell response (8) and in the current study by TRM cell-mediated response. Since *LmCen^-/-^* parasites do not induce any pathology, it could serve as a safer alternative to leishmanization. Therefore, immunization with *LmCen* ^-/-^ parasites may represent a more practical vaccination strategy with a realistic possibility of gaining approval for clinical use than leishmanization. In conclusion, this preclinical study in an animal model further confirms that *LmCen^-/-^* parasites should be explored as a potential *Leishmania* vaccine in future clinical trials.

## Author’s Note

The findings of this study are an informal communication and represent the authors’ own best judgments. These comments do not bind or obligate the Food and Drug Administration.

## Data Availability Statement

The original contributions presented in the study are included in the article/**Supplementary Material**. Further inquiries can be directed to the corresponding authors.

## Ethics Statement

The animal protocol for this study has been approved by the Institutional Animal Care and Use Committee at the Center for Biologics Evaluation and Research, US FDA (ASP 1995#26). In addition, the animal protocol is in full accordance with “The guide for the care and use of animals as described in the US Public Health Service policy on Humane Care and Use of Laboratory Animals 2015.”

## Author Contributions

NI, SK, PB, TS, and KT conducted the experiments and analyzed the data. NI, RD, and HLN designed the experiments. NI, SH, AS, GM, SG, RD, and HLN helped to write the article. All authors contributed to the article and approved the submitted version.

## Funding

This research was supported by the Global Health Innovative Technology Fund and CBER Intramural Research Program, FDA.

## Conflict of Interest

The FDA is currently a co-owner of two US patents that claim attenuated *Leishmania* species with the Centrin gene deletion (US7,887,812 and US 8,877,213).

The remaining authors declare that the research was conducted in the absence of any commercial or financial relationships that could be construed as a potential conflict of interest.

## Publisher’s Note

All claims expressed in this article are solely those of the authors and do not necessarily represent those of their affiliated organizations, or those of the publisher, the editors and the reviewers. Any product that may be evaluated in this article, or claim that may be made by its manufacturer, is not guaranteed or endorsed by the publisher.
