# Supplementary material for: Leishmania Major Centrin Gene-Deleted Parasites Generate Skin Resident Memory T-Cell Immune Response Analogous to Leishmanization
Source: Front Immunol. 2022 Mar 28;13:864031. doi: 10.3389/fimmu.2022.864031 (PMC8996177; doi:10.3389/fimmu.2022.864031)
Supplement: Supplementary file 1 [file Presentation_1.pdf]

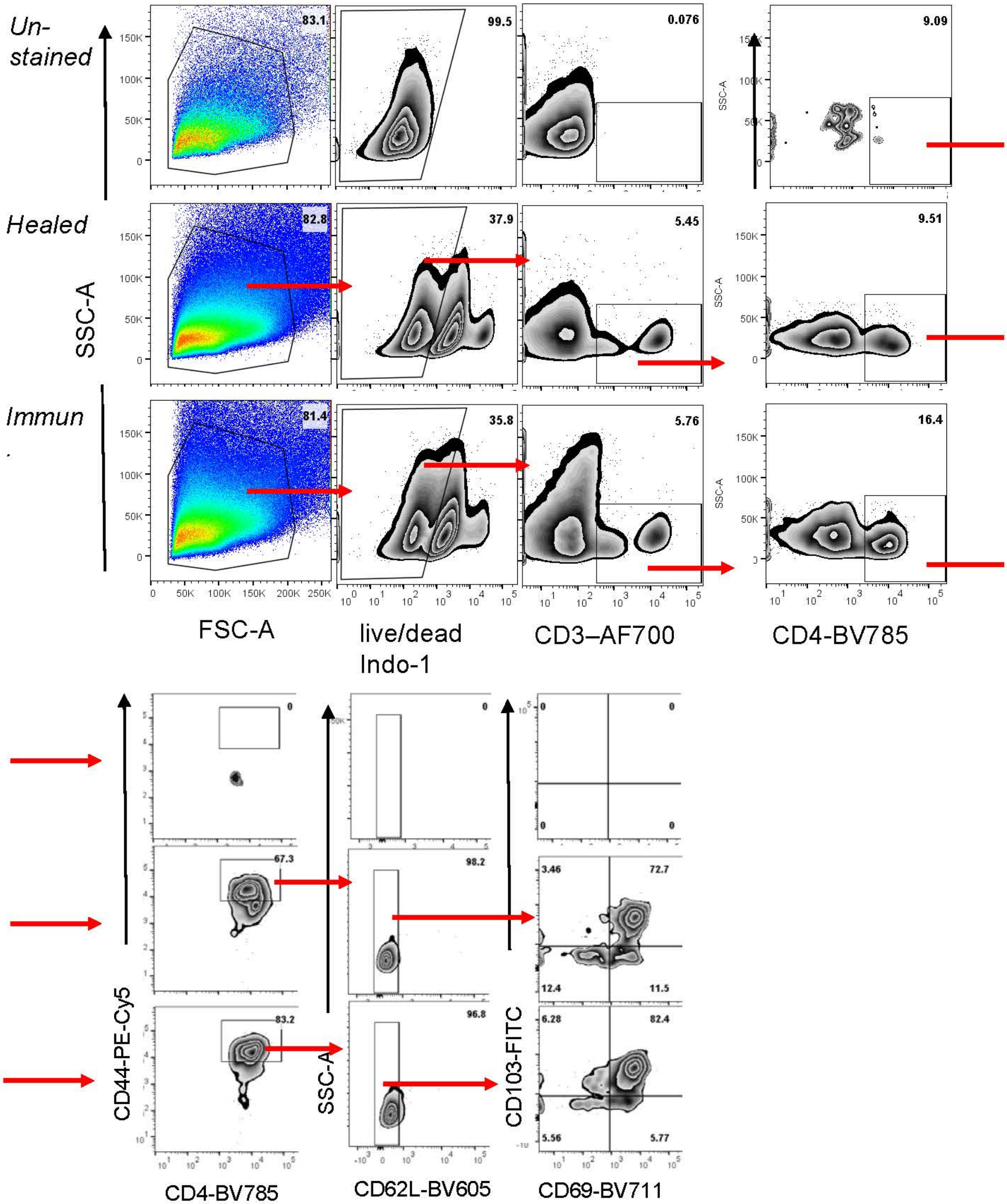

Supplementary Figure 1: Immunization with *LmCen*$^{-/-}$ generates CD4+ TRM cells in the skin. Mice were injected, intradermally, with either *LmCen*$^{-/-}$ or *L. major* wild type (*LmWT*). Baseline TRM population was measured in flank skin of non-immunized mice. **Gating strategy for Figure 1**. Data shown is from 15w post injection time point. Numbers in the quadrants are the proportion of the gated population of parent population.

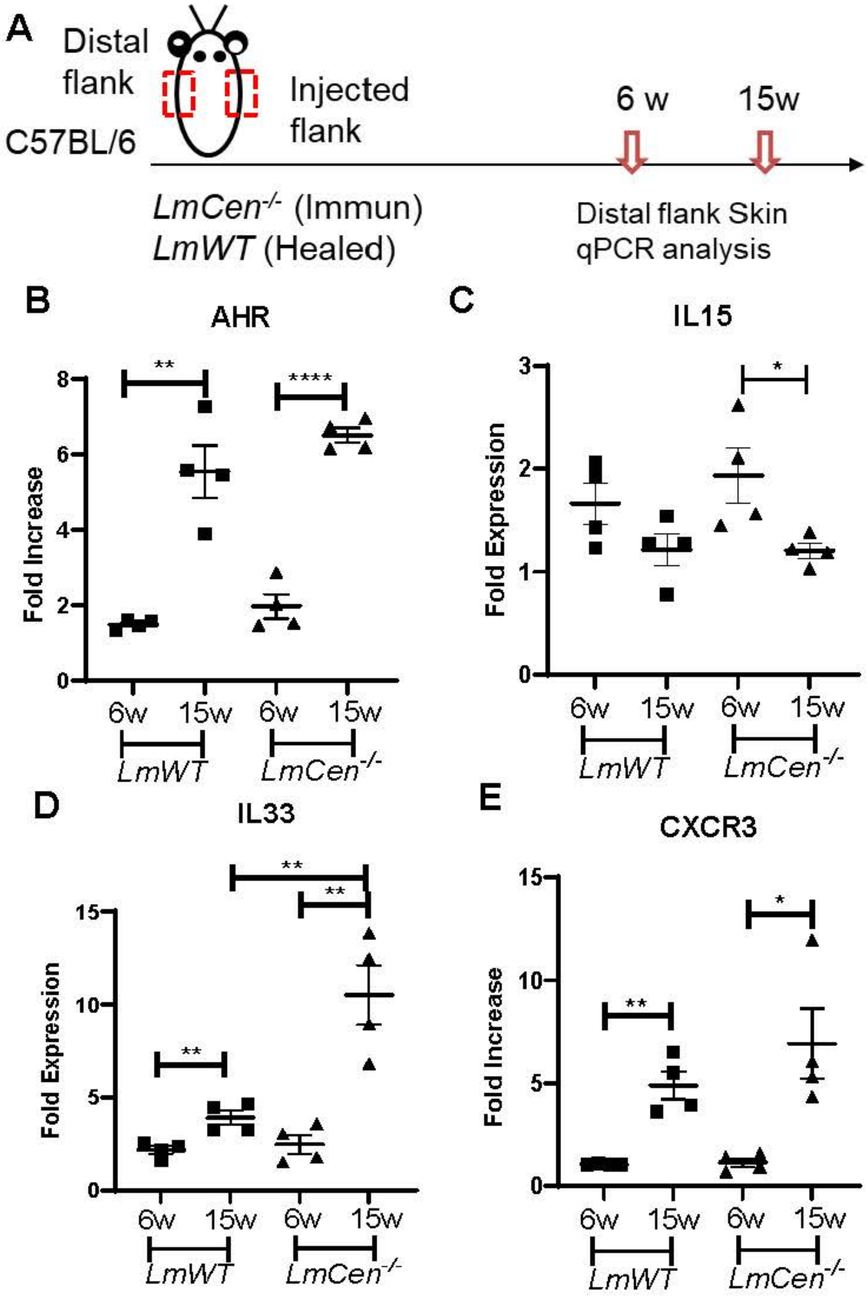

Supplementary Figure 2: Expression profile of cytokines and chemokine receptors supporting TRM cell generation at the distal flank skin. Mice were injected with *LmCen*$^{-/-}$ or *LmWT*, intradermally, in the right flank. The expression profile of indicated genes from the distal flank skin was assessed at 6- and 15- weeks post injection by qPCR. **(A)** Schematic plan of the experimental time points, injection and distal sites. **(B-E)** Expression of different transcripts, **(B)** AHR**, (C)** IL15**, (D)** IL33 and **(E)** CXCR3 at indicated time points. To determine the fold expression of each gene, 2-ΔΔCT method was employed. The data were normalized to GAPDH expression and shown as the fold change relative to age matched naïve mice. Results are representative of one independent experiments, repeated at least 2 times, with total 4 mice per group. Bars represent the means with SEM in each group. Statistical analysis was performed by unpaired two-tailed t test (*$p < 0.05$, **$p<0.009$, ****$p<0.00005$).

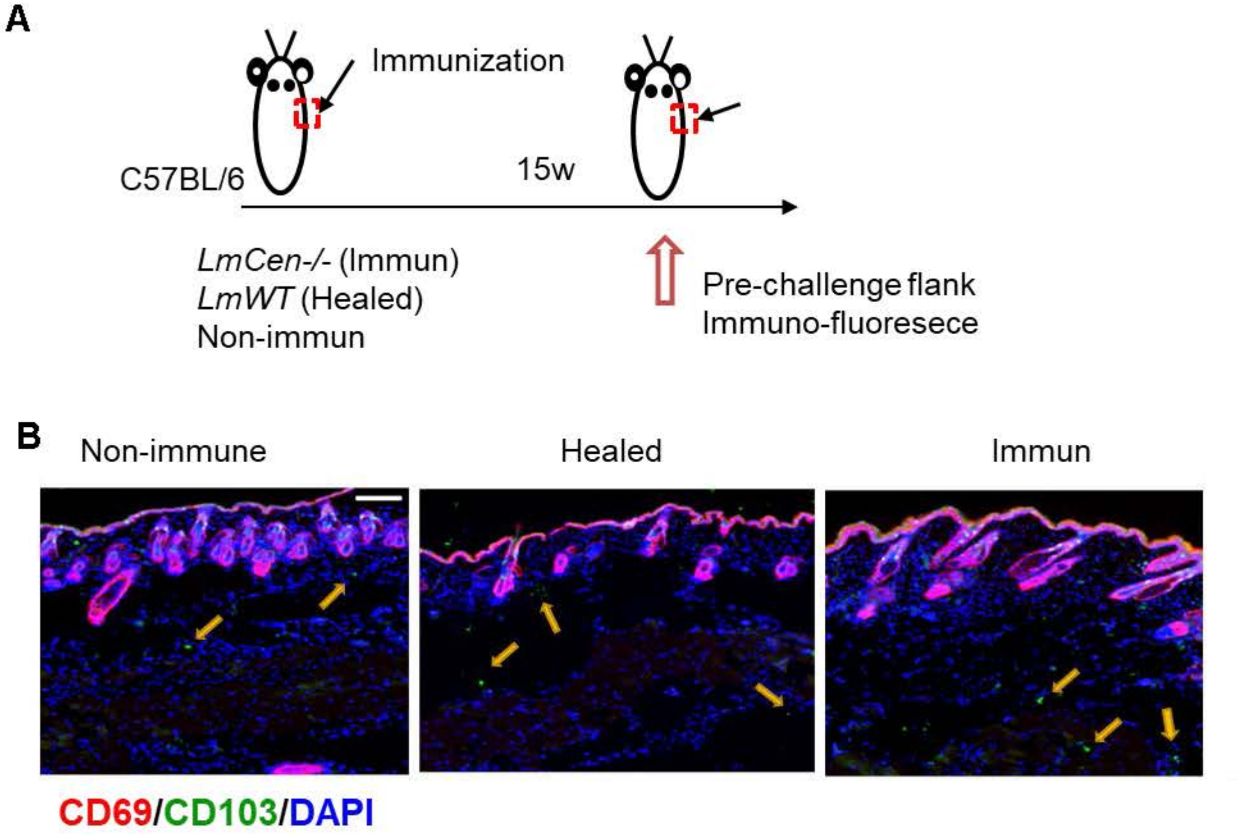

Supplementary Figure 3: Immunohistology of skin tissues from fifteen weeks non-immunized, healed and Immunized mice before challenge. TRM cells were analyzed by immunofluorescence staining. (A) Schematic plan of the experimental time point (B) A merged image of CD69 (red), CD103 (green) and DAPI (blue) in the flank skin of at the site of immunization, before challenge. Yellow arrows indicate CD103+ cells. Scale bar is 200μm. Results are representative of one of two independent experiment, with 3 mice per group.

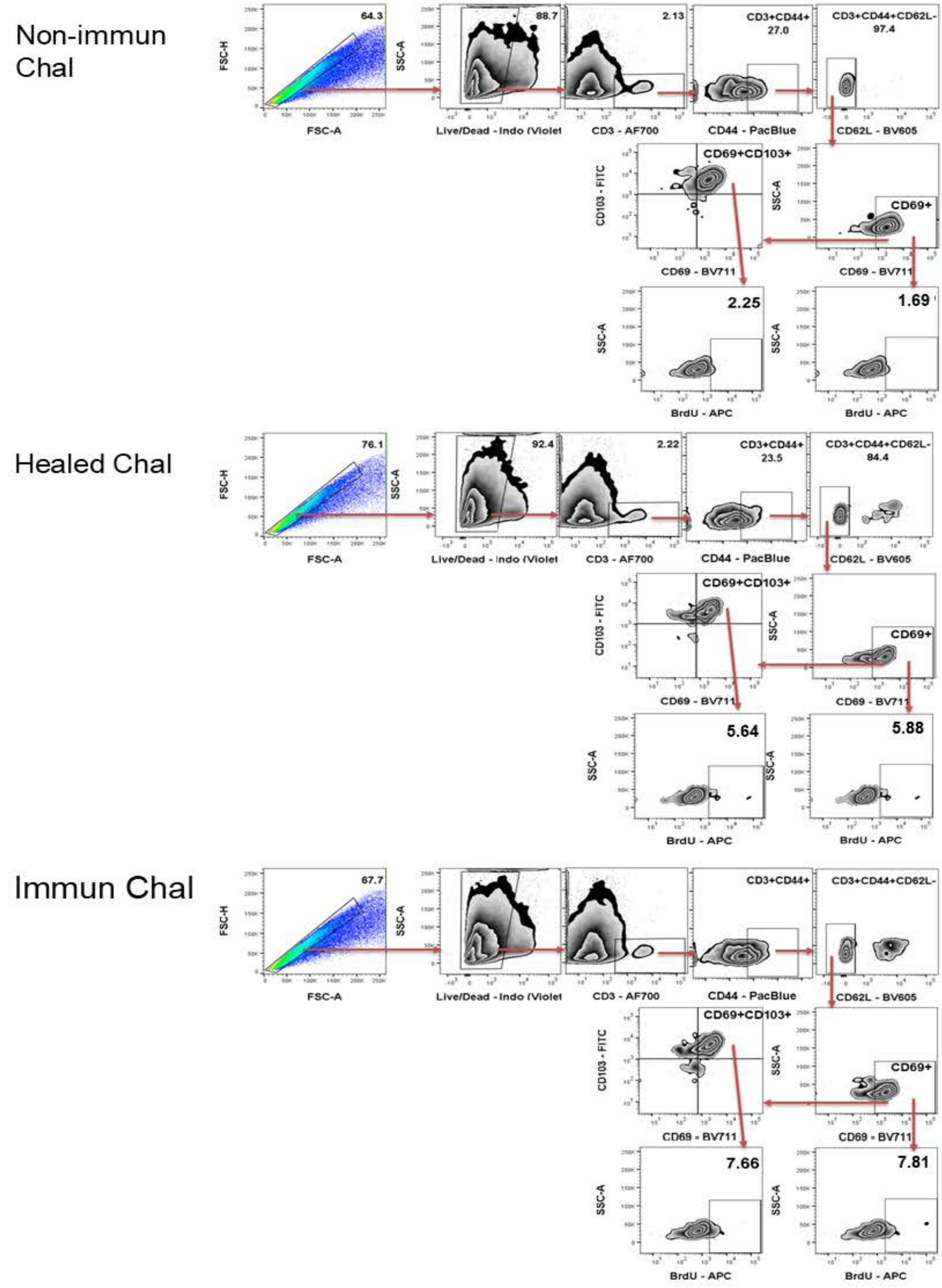

Supplementary Figure 4: TRM cells from *LmCen*$^{-/-}$ immunized mice proliferate locally following challenge with virulent *LmWT* parasites. Fifteen weeks immunized, and healed mice were challenged with *LmWT* parasites, in the flank skin and injected with BrdU, as described in the materials and method. Mice were euthanized, flank skins were collected 7 days post challenge and analyzed for BrdU positive cells. **Gating strategy for Figure 4**. The graphs are representative of the data combined from 2 independent experiments for 3 groups: non-immunized Challenged (Non-immun Chal) group, n=5; healed challenged (Healed Chal) group, n=6; and Immunized challenged (Immun Chal) group, n=6.

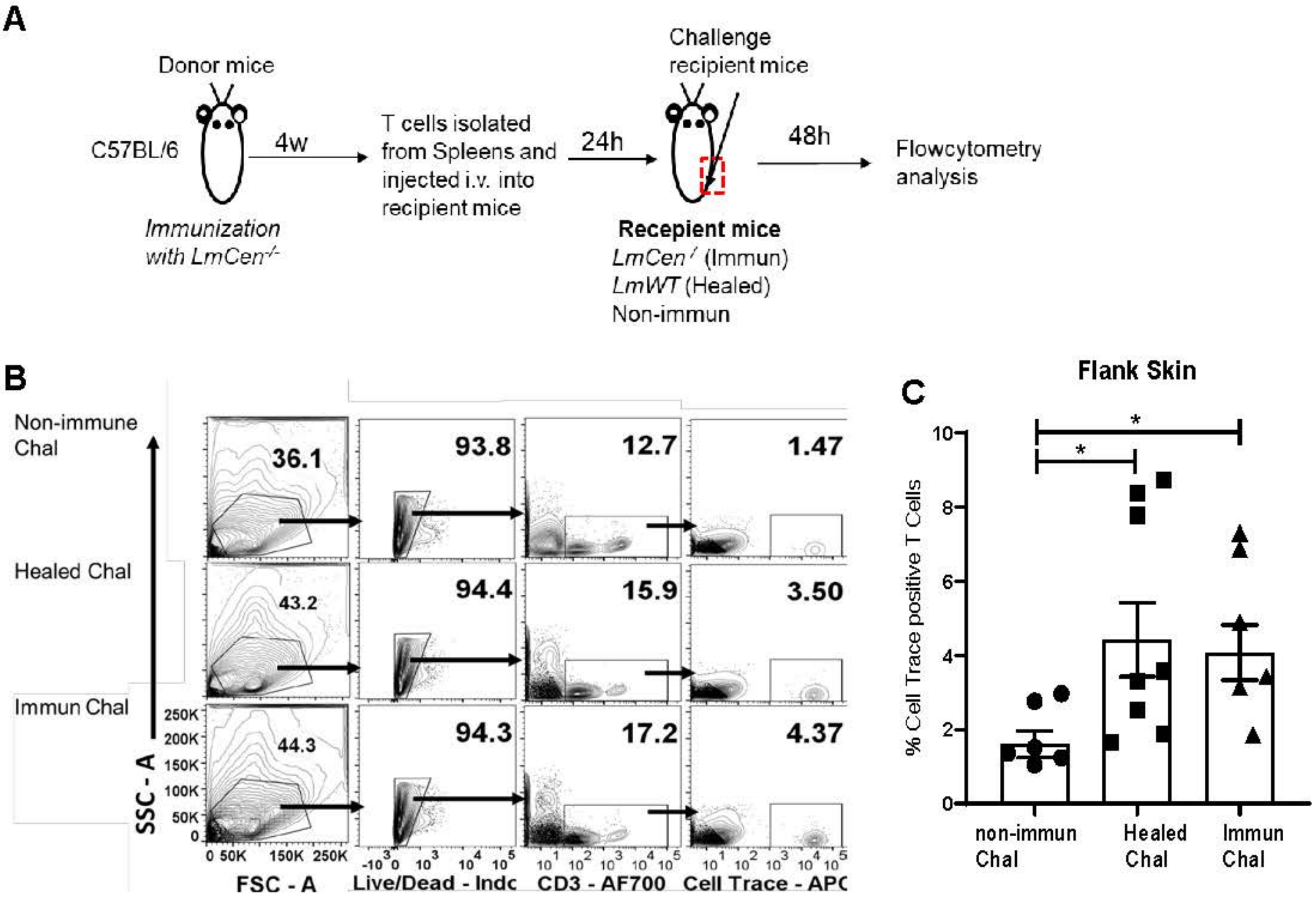

Supplementary Figure 5: Recruitment of circulatory T cells to the skin of *LmCen*$^{-/-}$ immunized mice after challenge. T cells from the spleens of *LmCen*$^{-/-}$ immunized mice were labeled with Far Red cell trace to track them after transfer. The cells were injected i.v. into naïve, healed or *LmCen*$^{-/-}$ immunized mice (15- week post infection). Recipient mice were then challenged with *L. major* WT parasites. 48h post challenge, skin were collected and cell recruitment to the challenge sites were analyzed by flow cytometry analysis for cell trace positive cells. **(A)** Experimental diagram. **(B)** Gating strategy representative flow plots of Far Red^{+} CD3^{+} T cells from the flank skin challenged mice. Numbers in the quadrant represents the percentage of the gated population of parent population **(C)** Frequency of transferred *LmCen*$^{-/-}$ immune CD3^{+} T cells in the flank skin of non-immune challenged, healed challenged or immune challenged mice 48h post challenge. Y axes represents the percentage of gated population of total skin CD3^{+} T cells. *$p<0.03$. Data shown is combined results from two independent experiments, n=6-8. Results are mean ± SEM, statistical analysis was performed by tow tailed unpaired t-test.

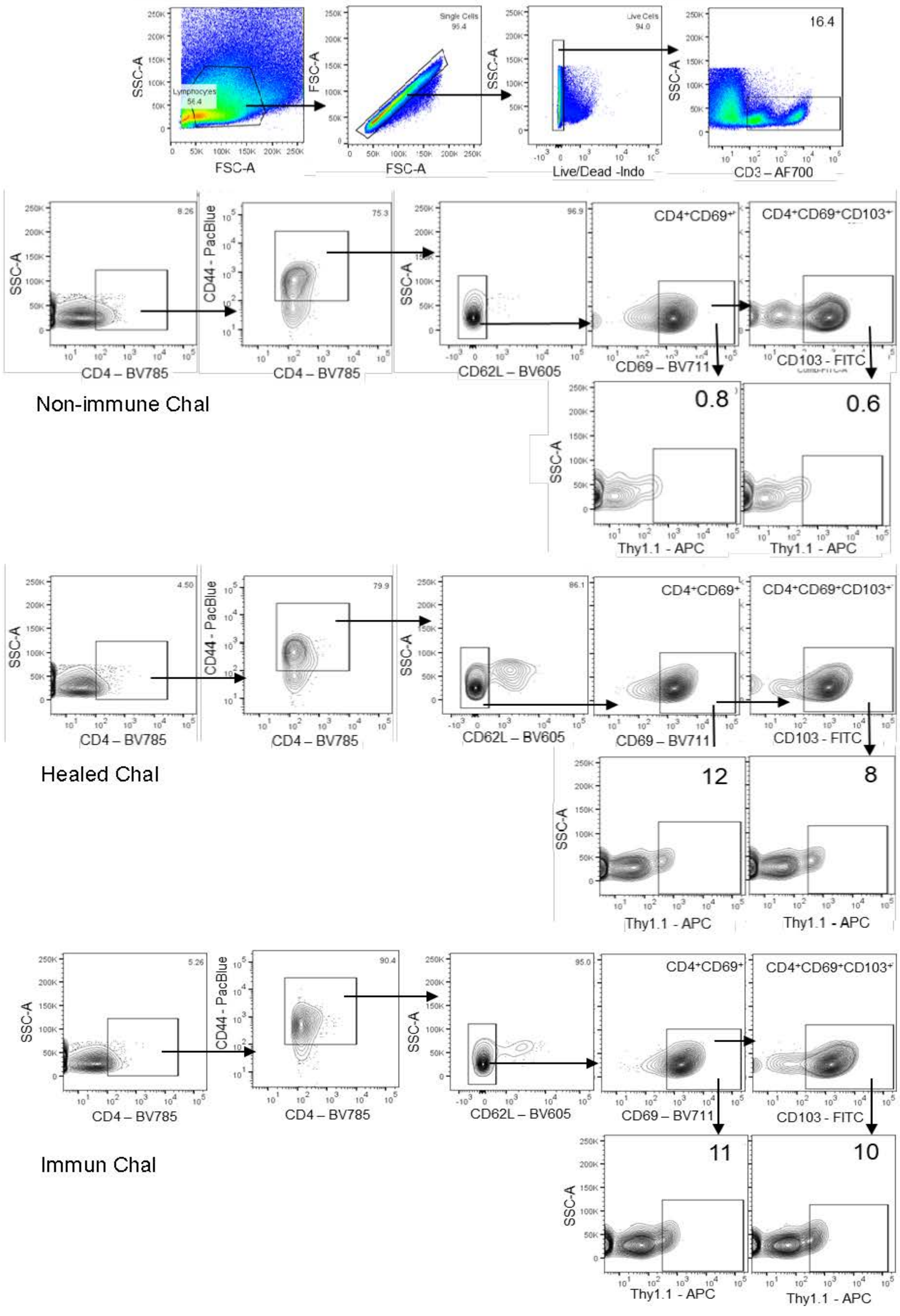

Supplementary Figure 6: *L. major* specific skin TRM cells produce IFNγ in response to *LmWT* challenge infection. Non-immunized, healed and immunized Ifnγ/Thy1.1 mice were challenged with *LmWT* parasite in the flank skin. T cells from challenged skin were isolated 5- days post challenge and cytokine production was assessed with flow cytometry analysis. **Gating strategy for Figure 5**. Gating strategy shown is representative of samples acquired. Numbers in the quadrants are the proportion of the gated population of $CD69^+$ population.
