# Supplementary material for: Leishmania Major Centrin Gene-Deleted Parasites Generate Skin Resident Memory T-Cell Immune Response Analogous to Leishmanization
Source: Front Immunol. 2022 Mar 28;13:864031. doi: 10.3389/fimmu.2022.864031 (PMC8996177; doi:10.3389/fimmu.2022.864031)
Supplement: Supplementary file 2 [file Table_1.docx]

Table S1. List of genes amplified from RNA extracted from mice skin after injection of *LmCen^-/-^* or *LmWT* parasites Related to main Figure 2.

Table-1

| **Target Gene** | **Source** | **Assay ID** |
| --- | --- | --- |
| *CXCR3* | Applied Biosystems | *Mm00438259_m1* |
| *CCR8* | Applied Biosystems | *Mm99999115* |
| *TGFB* | Applied Biosystems | *Mm01178819* |
| *IL15* | Applied Biosystems | *Mm00434210* |
| *IL33* | Applied Biosystems | *Mm00505403* |
| *AHR* | Applied Biosystems | *Mm00478932* |
| *GAPDH* | Applied Biosystems | *Mm99999915_g1* |
